# Hypoxaemia prevalence and its adverse clinical outcomes among children hospitalised with WHO-defined severe pneumonia in Bangladesh

**DOI:** 10.7189/jogh.11.04053

**Published:** 2021-09-11

**Authors:** Ahmed Ehsanur Rahman, Aniqa Tasnim Hossain, Mohammod Jobayer Chisti, David H Dockrell, Harish Nair, Shams El Arifeen, Harry Campbell

**Affiliations:** 1 The Usher Institute, Edinburgh Medical School: Molecular, Genetic and Population Health Sciences, The University of Edinburgh, Edinburgh, UK; 2 Maternal and Child Health Division, International Centre for Diarrhoeal Disease Research (icddr,b), Dhaka, Bangladesh; 3 Nutrition and Clinical Services Division, International Centre for Diarrhoeal Disease Research (icddr,b), Dhaka, Bangladesh

## Abstract

**Background:**

With an estimated 1 million cases per year, pneumonia accounts for 15% of all under-five deaths globally, and hypoxaemia is one of the strongest predictors of mortality. Most of these deaths are preventable and occur in low- and middle-income countries. Bangladesh is among the six high burden countries with an estimated 4 million pneumonia episodes annually. There is a gap in updated evidence on the prevalence of hypoxaemia among children with severe pneumonia in high burden countries, including Bangladesh.

**Methods:**

We conducted a secondary analysis of data obtained from icddr,b-Dhaka Hospital, a secondary level referral hospital located in Dhaka, Bangladesh. We included 2646 children aged 2-59 months admitted with WHO-defined severe pneumonia during 2014-17. The primary outcome of interest was hypoxaemia, defined as SpO_2_ < 90% on admission. The secondary outcome of interest was adverse clinical outcomes defined as deaths during hospital stay or referral to higher-level facilities due to clinical deterioration.

**Results:**

On admission, the prevalence of hypoxaemia among children hospitalised with severe pneumonia was 40%. The odds of hypoxaemia were higher among females (adjusted Odds ratio AOR = 1.44; 95% confidence interval CI = 1.22-1.71) and those with a history of cough or difficulty in breathing for 0-48 hours before admission (AOR = 1.61; 95% CI = 1.28-2.02). Among all children with severe pneumonia, 6% died during the hospital stay, and 9% were referred to higher-level facilities due to clinical deterioration. Hypoxaemia was the strongest predictor of mortality (AOR = 11.08; 95% CI = 7.28-16.87) and referral (AOR = 5.94; 95% CI = 4.31-17) among other factors such as age, sex, history of fever and cough or difficulty in breathing, and severe acute malnutrition. Among those who survived, the median duration of hospital stay was 7 (IQR = 4-11) days in the hypoxaemic group and 6 (IQR = 4-9) days in the non-hypoxaemic group, and the difference was significant at *P* < 0.001.

**Conclusions:**

The high burden of hypoxaemia and its clinical outcomes call for urgent attention to promote oxygen security in low resource settings like Bangladesh. The availability of pulse oximetry for rapid identification and an effective oxygen delivery system for immediate correction should be ensured for averting many preventable deaths.

Pneumonia accounts for approximately 15% of all under-5 deaths, making it one of the leading causes of mortality in this age group globally [1]. Most of these deaths are preventable and occur in low- and middle-income countries (LMICs) [2,3]. Considering the high morbidity and mortality burden, the World Health Organisation (WHO) and UNICEF declared it the major “forgotten killer of children” [4].

Hypoxaemia, defined as low oxygen saturation in arterial blood (peripheral capillary oxygen saturation (SpO_2_)<90%) by the World Health Organization, is a common complication of pneumonia and other acute lower respiratory infections (ALRIs) with wide variations in prevalence across different clinical severity classifications and geographical areas [5,6]. Hypoxaemia is one of the strongest predictors of mortality among children suffering from pneumonia and other ALRIs [7,8]. Most of the hypoxaemia-related deaths can be prevented by routine assessment, early detection, and rapid correction through appropriate oxygen therapy [9,10].

Bangladesh is one of six high burden countries with an estimated 4 million episodes of clinical pneumonia and 677 000 episodes of severe pneumonia annually among children under-5 years of age [11]. Like many other LMICs, pneumonia is the primary killer among this age group causing approximately 18% of all under-5 deaths in Bangladesh [12]. Therefore, in addition to achieving high coverage of Pneumococcal vaccine and *Haemophilus influenzae* Type b for prevention and ensuring appropriate antibiotic therapy for treatment, timely management of hypoxaemia is essential to avert these preventable deaths and achieve the 2025 Global Action Plan for Pneumonia and Diarrhoea (GAPPD) target [13-15].

WHO recommends routine assessment of hypoxaemia at the first point of contact and ensuring oxygen therapy in inpatient care, but there are significant gaps between policy and actual clinical practices [16,17]. In Bangladesh, very few public and private hospitals have the provision for routine assessment of hypoxaemia among children presenting with pneumonia or other ALRIs [18]. Hence, there are scant data regarding the current prevalence of hypoxaemia among children with pneumonia. This paper aims to assess the prevalence of hypoxaemia, disaggregated by background characteristics and clinical history, among children hospitalised with WHO-defined severe pneumonia in a non-government hospital in Bangladesh. We also explore the contribution of hypoxaemia as a predictor of death and referral due to clinical deterioration.

## METHODS

**Study design:** We conducted a secondary analysis of data obtained from icddr,b-Dhaka Hospital, a non-government hospital located in Dhaka, Bangladesh's capital.

**Study settings:** The icddr,b-Dhaka Hospital provides treatment to approximately 200 000 patients annually, of which more than half are children under-5 years of age [19]. Most of these patients are from a low-socioeconomic background, principally residing in urban or peri-urban Dhaka. Diarrhoea is the primary entry point for admission to icddr,b-Dhaka Hospital; however, children with cough and respiratory distress receive treatment irrespective of their diarrhoea status. Most patients are treated through the short stay ward (less than 24 hours). They are admitted to the special care ward (SCW) if severe complications, including pneumonia and other ALRI. The SCW of icddr,b treats on average 1200 under-five children per year, and among which almost 50% are severe pneumonia cases. There are nine fixed beds in SCW. Extra beds are provided for sick children when there is a surge of severe patients, including pneumonia. This SCW has mechanical ventilators, non-invasive ventilation, piped oxygen, cardiac monitors, pulse oximetry, infusion pumps, syringe pumps, and inotropes and vasopressor drugs. All children with severe pneumonia and hypoxemia used to receive (since August 2013) locally made low-cost bubble-continuous positive airway pressure (CPAP) as a part of the hospital standard of care.

**Participants:** In this secondary analysis, we included all children aged 2-59 months admitted to the SCW with WHO-defined severe pneumonia between 2014 and 2017.

**Measurements:** All children admitted to SCW were seen by the attending physician, who performed a physical examination. The hospital on-duty nurses collected the background characteristics (including age) and the history of illness based on a standard form as a part of routine practice. In addition, they also measured the height and weight on admission. WHO reference value was used to calculate the z-score and classify the malnutrition level. The attending nurse also measured SpO_2_ for all children presenting with cough and difficulty in breathing on admission. A portable pulse oximetry (OxiMax N-600; Nellcor, Boulder, CO, USA) with a paediatric probe was used for SpO_2_ assessment. The clinical definition of the 2013 version of the WHO Pocketbook of Hospital Care for Children was used for pneumonia classification [20]. Severe pneumonia was defined as children with a history of cough and difficulty in breathing with central cyanosis or SpO_2_ < 90% or severe respiratory distress defined as grunting or very severe chest indrawing or signs of pneumonia according to WHO Pocket Book of Hospital Care for Children: Guidelines for the Management of Common Childhood Illnesses [20] (≥50 breaths per minute in a child aged 2-11 months, and ≥40 breaths per minute in a child aged 12-59 months), with any of the following danger signs: lethargy, unable to breastfeed or drink, convulsion, loss of consciousness. We extracted data from electronic individual patient records and included all children with WHO-defined severe pneumonia for this analysis.

**Patient management for pneumonia:** Children admitted to the SCW with severe pneumonia received antibiotics and supportive care following the WHO-pocket book guidelines [20]. Moreover, they received frequent monitoring by the clinical team and nutritional support (breast milk, micronutrients, zinc). In addition, all children with hypoxaemia received O_2_ supplementation through nasal prongs using the locally made bubble CPAP [21].

**Data Analysis:** We used Stata version 14.0 [22] for data analysis. The primary outcome of interest was hypoxaemia on admission, and we used the WHO recommended cut-off of SpO_2_ < 90% [20,23]. The secondary outcome of interest was adverse clinical outcomes defined as deaths during hospital stay or referral to higher-level facilities due to clinical deterioration. We used descriptive statistics (proportion with 95% confidence intervals) to report the prevalence of hypoxaemia, which was disaggregated by background characteristics (age and sex), history of illness (number of days suffering from fever and cough or difficulty in breathing before admission) and presence of comorbidities (severe acute malnutrition- as<−3z score of weight for age). We also presented the seasonal variations in hypoxaemia prevalence by quarter. The average annual rate of increase was estimated using the following equation: r = [ln(p2/p1)]/(t2-t1), where t1 is the first time point, t2 is the last time point, p1 is prevalence in t1 and p2 is prevalence in t2. We used multiple logistic regression to present the relationship of different risk factors (background characteristics such as age and sex, history of illness and comorbidities) with hypoxaemia status after adjusting for the effect of potential confounders and covariates. We followed a similar approach to present the relationship between hypoxaemia status and adverse hospital outcomes (death during hospitalisation or referral to higher-level facilities due to clinical deterioration separately and a composite indicator). The risks were presented with adjusted odds ratios (AORs) with 95% confidence intervals (CI). Among those who were alive at the end of their hospital stay, we estimated the median duration of hospital stay with the inter-quartile range (IQR). Those who died during the hospital stay or left the hospital against medical advice were treated as missing values for this analysis. The difference in the median duration of hospital stay by their hypoxaemia status on admission was assessed using the nonparametric equality of medians test since they were not normally distributed. Shapiro-Wilk and Shapiro-Francia tests were used for testing the normality of distribution.

### Ethical considerations

We obtained routine health data from icddr,b-Dhaka hospital's patient records. The researchers were not directly involved with services and did not have any interaction with the patients during the data collection. Ethical approval for this study was obtained from the Institutional Review Board of icddr,b (PR-18054).

## RESULTS

A total of 2646 children met the inclusion criteria, and all were included in the analysis. **Table 1** presents the background characteristics of the children who were included in this analysis. Around 70% of children were aged 2-11 months, and 65% were male. Approximately 60% of them had a history of fever, and 25% had a history of difficulty in breathing before admission. Around 42% of them had severe acute malnutrition.

**Table 1:** Background characteristics of children aged 2-59 months admitted to icddr,b-Dhaka Hospital with WHO-defined severe pneumonia between 2014-17, N = 2646

	N	%
**Age (months):**		
2-11	1895	72
12-59	751	28
**Sex:**		
Male	1707	65
Female	939	35
**History of fever:**		
No fever	1101	42
0-1 days	239	9
2-6 days	1040	39
7 or more days	266	10
**History of cough or difficulty in breathing:**		
No respiratory distress	1949	74
0-48 h	384	14
More than 48 h	313	12
**Severe acute malnutrition:**		
No	1116	42
Yes	1530	58
Total	2646	100

**Figure 1:**
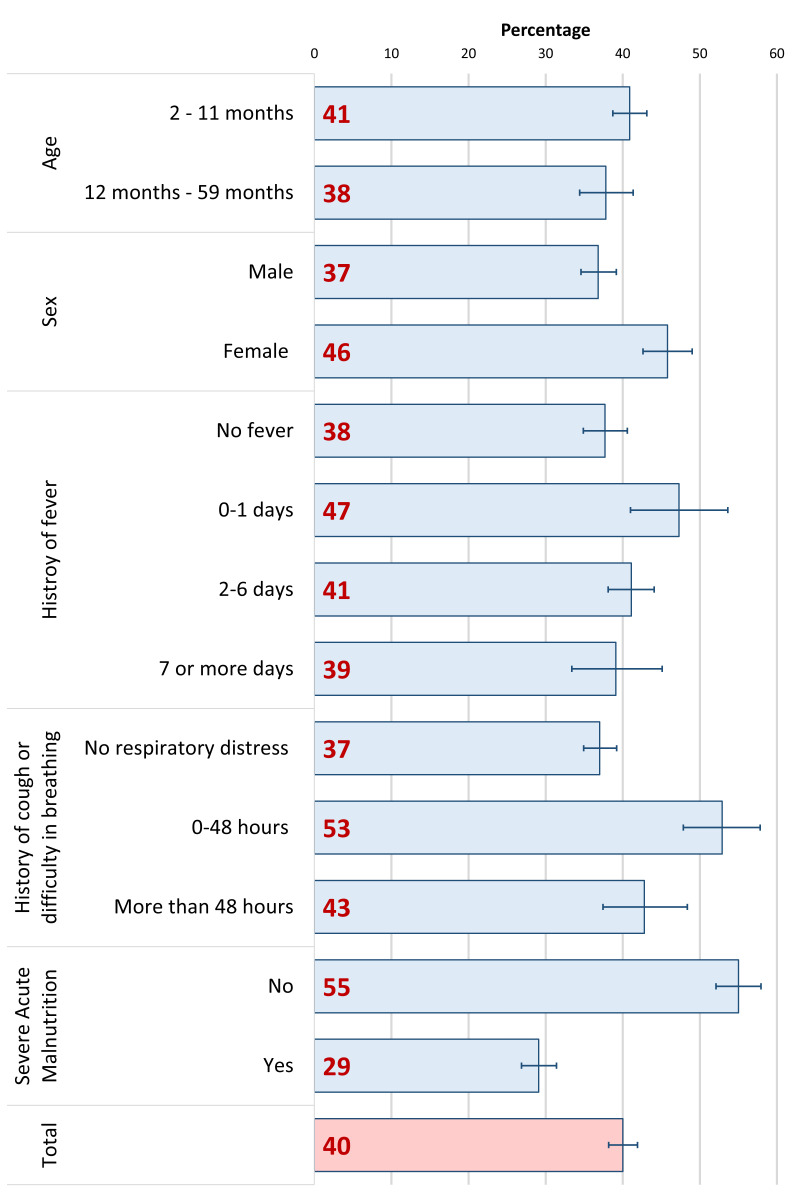
Hypoxaemia prevalence among children aged 2-59 months admitted to icddr,b Dhaka Hospital with WHO-defined severe pneumonia between 2014-17, presented in percentage by background characteristics; N = 2646.

**Figure 1** represents the hypoxaemia prevalence disaggregated by background characteristics, clinical history, and comorbidities. On admission, the overall prevalence was 40% (95% CI = 39%-43%). The prevalence was higher among females (46%; 95% CI = 43%-49%) and those who had a history of fever for 0-1 day (47%; 95% CI = 42%-54%) and cough or difficulty in breathing for 0-48 hours (53%, 95% CI = 48%-58%) before admission. Although not statistically significant, the prevalence of hypoxaemia was somewhat higher among the younger age group than that of older children (Figure S1 in the **Online Supplementary Document**)

**Figure 2:**
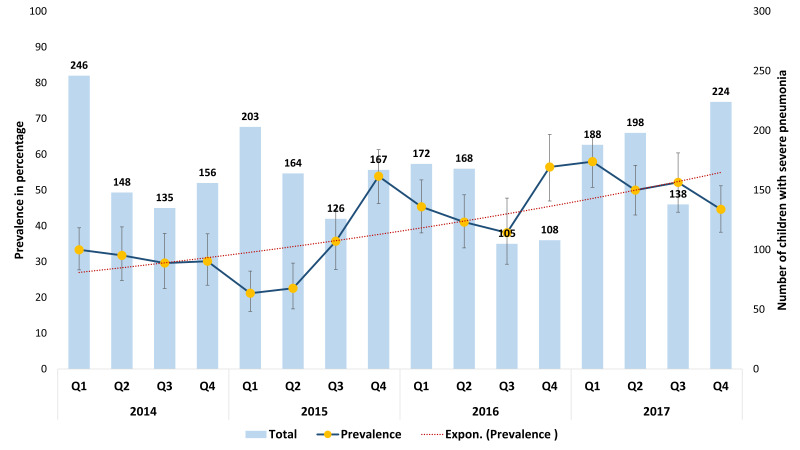
Hypoxaemia prevalence among children aged 2-59 months admitted to icddr,b Dhaka Hospital with WHO-defined severe pneumonia between 2014-17, presented in percentage by quarters; N = 2646.

**Figure 2** represents the seasonal variation in hypoxaemia prevalence between 2014 and 2017. Here the prevalence (%) is presented with the line-graph, and the number of children hospitalised with severe pneumonia is presented with the column-graphs. Although no notable seasonal pattern was observed, the average annual prevalence increased from 32% in 2014 to 51% in 2018. The average annual rate of increase was around 19%.

**Table 2** represents the relationship of various risk factors with the hypoxaemia status of children. The odds of hypoxaemia were higher among females (AOR = 1.44; 95% CI = 1.22-1.71). It was also higher among children who had a history of cough or difficulty in breathing for 0-48 hours on admission (AOR = 1.61; 95% CI = 1.28-2.02).

**Table 2:** Association between various risk factors and hypoxaemia status of children aged 2-59 months admitted to icddr,b-Dhaka Hospital with WHO-defined severe pneumonia between 2014-17, presented in odds ratio and adjusted odds ratio, N = 2646

	% (with hypoxaemia)	OR (CI)	AOR (CI)
**Age:**			
2-11 months	41	Reference	Reference
12-59 months	38	0.88 (0.74,1.05)	0.85 (0.71,1.02)
**Sex:**			
Male	37	Reference	Reference
Female	46	1.45 (1.23,1.7)	1.44 (1.22,1.71)
**History of fever:**			
No fever	38	Reference	Reference
0-1 days	47	1.48 (1.12,1.96)	1.3 (0.97,1.75)
2-6 days	41	1.15 (0.97,1.37)	1.04 (0.87,1.25)
7 or more days	39	1.06 (0.81,1.4)	1.1 (0.82,1.46)
**History of cough or difficulty in breathing:**			
No respiratory distress	37	Reference	Reference
0-48 h	53	1.91 (1.53,2.38)	1.61 (1.28,2.02)
More than 48 h	43	1.27 (1,1.62)	1.1 (0.86,1.42)
**Severe acute malnutrition:**			
No	55	Reference	Reference
Yes	29	0.34 (0.29,0.39)	0.35 (0.3,0.41)
**Total**	40		

OR – odds ratio, CR – confidence interval, AOR – adjusted odds ratio

Among all children admitted with WHO-defined severe pneumonia, 79% were discharged with medical advice, 6% died during admission, 9% were referred to a higher-level facility due to clinical deterioration, and another 6% left the hospital against medical advice. Among the children with hypoxaemia, a higher proportion (13%) died during the hospital stay or referred to a higher-level facility (17%), in comparison to those who did not have hypoxaemia (dead 2%, referred 4%) (Supplementary Figure 2). The overall case-fatality rate was 7.5% over the study period, which was significantly (*P* < 0.01) higher among the children who had hypoxaemia on admission (17.1%), compared to those who did not (2.1%). Supplementary Figure 3 present the trend in case-fatality rate by years.

**Figure 3:**
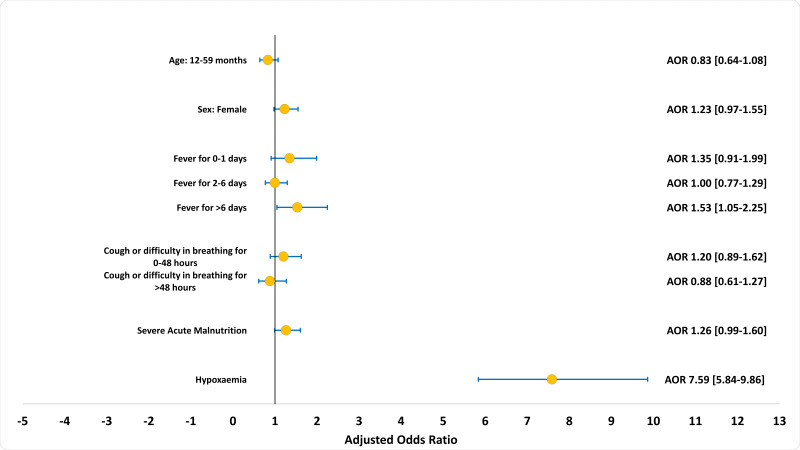
Association of adverse clinical outcomes with hypoxaemia, other background characteristics and history of illness among children aged 2-59 months admitted to icddr,b Dhaka Hospital with WHO-defined severe pneumonia between 2014-17, presented in adjusted *odds ratio*; N = 2646.

**Figure 3** presents the association between hypoxaemia status and adverse clinical outcomes (death or referrals to a higher-level facility due to deterioration in the patient's condition). Hypoxaemia is the strongest predictor of adverse clinical outcomes (AOR = 7.59; 95% CI = 5.84-9.86). History of fever for more than 7 days (AOR = 1.53; 95% CI = 1.05-2.25) was another significant predictor of adverse outcomes. Hypoxaemia was also the strongest predictor of mortality (AOR = 11.08; 95% CI = 7.28-16.87) and referral (AOR = 5.94; 95% CI = 4.31-17) independently. The details of the multivariable regression model are presented in Tables S1-S3 in the **Online Supplementary Document**).

**Figure 4:**
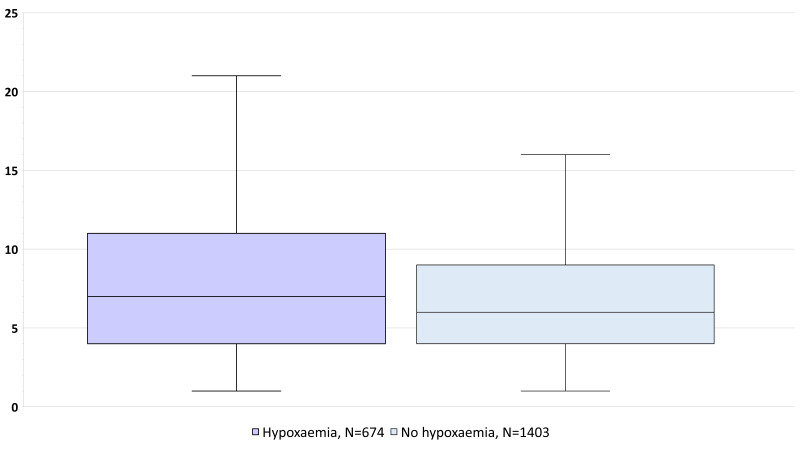
Box plot of the duration of hospital stay by hypoxaemia status on admission among those who were alive at the end of hospital stay (N = 2646).

**Figure 4** presents the difference in median duration for hospital stay by hypoxaemia status on admission. The median duration was 7 (IQR 4-11) days among those who had hypoxaemia and 5 (IQR 4-9) days among those who did not have hypoxaemia. The difference was significant at *P* < 0.001.

## DISCUSSION

We found that the prevalence of hypoxaemia among children hospitalised with severe pneumonia was 40% on admission. The odds of hypoxaemia were higher among females and those with a history of cough or difficulty in breathing. Hypoxaemia was the strongest predictor of death or referral to a higher-level facility among other covariates such as age, sex, history of fever and cough or difficulty in breathing and malnutrition. The median duration of hospital stay was significantly higher in the hypoxaemic group than in the non-hypoxaemic group.

Hypoxaemia is common among children with pneumonia and requires aggressive correction with oxygen therapy to prevent mortality and long-term consequences. Data to derive the local prevalence of hypoxaemia and its adverse consequences are crucial for the health care providers to appreciate the importance of routine assessment of hypoxaemia and for policymakers to prioritise relevant interventions in programme planning. We report the most up-to-date estimate of the prevalence of hypoxaemia and its adverse clinical outcomes in Bangladesh.

The hypoxaemia prevalence (40%) reported in our study is somewhat similar to the systematic review conducted by Lozano et al. in 2001 but much higher than the one conducted by Subhi et al. in 2009 [5,6]. However, the 2009 review also reported wide variations between clinical severity classifications as the prevalence of hypoxaemia ranged from 9% to 39% among pneumonia cases in Asia. Moreover, the 2009-review followed the pre-2013 WHO-classification, including chest indrawing as a sign of severe pneumonia. Chest indrawing is a common sign of pneumonia but a relatively weak predictor of its clinical severity [24-26]. On the contrary, we included only hospitalised children with severe pneumonia and followed the 2013 WHO-classification, which dropped chest indrawing as a sign of severe pneumonia [20]. Moreover, the hypoxaemia prevalence reported in this paper is within the ranges of several other conducted in some South-Asian (17% to 57%) and African countries (18% to 57%) and published after 2009 [27-33].

The hypoxaemia prevalence reported in this paper is based on children admitted in icddr,b-Dhaka hospital, a non-government hospital located in Bangladesh's capital. We acknowledge that the results are not representative of all types (public and private) and levels (health centre, primary, secondary, and tertiary) of health facilities in Bangladesh. However, icddr,b mainly deals with primary cases of pneumonia and serves as the primary point for contact for severe and relatively complicated cases to its catchment population due to its good reputation for pneumonia care in the catchment communities. It provides all services free of cost, and the majority of patients receiving care from icddr,b-Dhaka hospital is from a low-socioeconomic background. Hence, it is comparable to the district- and upazila- (sub-district level public hospitals where people with access to less financial resources predominately seek free-of-cost services. Another important consideration is the issue of selection bias. Although the icddr,b-Dhaka hospital is specialised for treating diarrhoea, all children meeting the criteria for hospitalisation based on the WHO-pneumonia-classifications were offered admission irrespective of their diarrhoea status and other comorbid conditions [20]. All eligible children admitted to the special care ward were included in this analysis. Hence, we are confident that the prevalence estimate is not majorly influenced by selection bias. Lastly, the accuracy and reliability of hypoxaemia measurements are critical to the internal validity of the relevant estimates. In our study, SpO_2_ assessment was included as a part of the routine assessment on admission, and trained nurses measured the SpO_2_ status with handheld pulse oximetry with an appropriate paediatric probe. The clinical assessment and physical examination were conducted by trained doctors, which added additional value to the validity of relevant measurements. Moreover, our study was conducted immediately after completing the randomised control trial on bubble CPAP in icddr,b-Dhaka hospital [21]. Therefore, the knowledge, skill, and behaviour of the clinical team responsible for the management of pneumonia were optimum, resulting in almost universal assessment and documentation of hypoxaemia status among all children admitted with a history of cough or difficulty in breathing. It explains the unusually low level of data missingness regarding hypoxaemia status on admission in our study.

We found that hypoxaemia on admission was associated with a short history of cough or difficulty in breathing, as reported by the parents. The association was less strong with a relatively long history. The severity of complications on admission can explain this. Parents are more likely to seek care from a hospital with a short history of acute respiratory complaints if they perceive it as more severe or the condition deteriorates rapidly. Gender preference in care-seeking practices can explain the difference observed in hypoxaemia prevalence among boys and girls. A survey with a nationally representative sample in Bangladesh found that boys were more likely than girls to be taken to a health facility or provider (46% and 31%, respectively) for acute respiratory infections [12]. Similarly, other studies based in hospital settings demonstrated a sex-based disparity in the severity of pneumonia and deaths among children admitted to hospitals in Bangladesh [34-37]. Therefore, it is highly likely that the female children included in our analysis were brought to the hospital in a more critical stage than their male counterparts. We found that hypoxaemia on admission was strongly associated with the nutritional status. Although we found a negative association, another study conducted in similar settings did not find any significant associations between nutritional status and hypoxaemia [38].

We present the hypoxaemia prevalence disaggregated by 16 quarters between 2014 and 2017. Although we did not find any notable seasonal variation, the hypoxaemia prevalence increased at an average annual rate of around 19% during this period. The introduction of PCV and Hib vaccine in the national immunisation programmes in several countries, including Bangladesh, resulted in a significant reduction in bacterial pneumonia, which has consequently made RSV and other viruses the common pathogens causing pneumonia among children [39-43]. This change in the pneumonia aetiology may shift clinical features, including hypoxaemia, as approximately 20% of the hospitalised children with respiratory syncytial virus-acute lower respiratory infections (RSV-ALRI) have hypoxaemia [44]. This may explain the change in hypoxaemia prevalence that we observed during the study duration. Another potential explanation could be the pneumonia care-seeking practice in Bangladesh. According to the Bangladesh Demographic and Health surveys, care-seeking for parents reported acute respiratory infection from the medically trained provider had increased from 20% in 2004 to 40% in 2017, implying that the parents are more aware of pneumonia-related symptoms and complications [12]. Hence, appropriate care-seeking practices from health facilities and formal health care providers may have contributed to better capturing the more complicated cases, including hypoxaemia, through hospital-based assessments.

There is significant debate regarding clinical prognostic factors, in addition to SpO_2_ status, that can be used in routine care to assess the severity of pneumonia and mortality [7,45]. Our analysis also found that several background characteristics, clinical history, and other comorbid conditions such as severe acute malnutrition are associated with hypoxaemia status on admission. This finding is consistent with another Gambian study which reported a rapid respiratory rate, grunting, and absence of a history of fever were the best independent predictors of hypoxaemia among children aged 2-33 months [46]. Further studies are necessary to draw insights into the association between hypoxaemia and severe acute malnutrition. However, it is challenging to develop a predictive model based on these signs and symptoms, which can reliably and accurately identify hypoxaemia. A systematic review reported that several clinical signs alone or in combination have high sensitivity but very low specificity of predicting hypoxaemia, which substantially minimises its usability in clinical settings [47]. There are also major issues regarding the validity and reliability of clinical assessment by health service providers [48-51]. Regarding the recall of pneumonia-related clinical symptoms by caregivers, both the sensitivity and specificity were poor [52].

In our study, the case fatality rate of children hospitalised with severe pneumonia was around 7.5%. A large retrospective observational study in Malawi estimated the case fatality rate of 6.6% based on more than 100 000 hospitalised pneumonia cases from 40 hospitals [53]. Another study conducted in a multi-speciality referral hospital in Chennai also reported a case fatality rate of around 8% among clinical pneumonia cases [54]. Although the rates reported in these two studies are similar to the rate reported in our study, there is a potential difference in the case-mix. The Malawian study used the pre-2013 WHO-pneumonia classification, which included both severe and non-severe cases, whereas we used the 2013 WHO-pneumonia classification. The Malawian study also reported a higher case fatality rate (12%) among children with very severe pneumonia than that of our study. This may be because Malawi has a high burden of HIV. Moreover, after the successful bubble CPAP trial, icddr,b-Dhaka hospital introduced routine assessment of hypoxaemia on admission and use of bubble CPAP for hypoxaemia management as the standard of care [21]. The overall clinical knowledge and pneumonia management skills of icddr,b-Dhaka hospital staff also improved due to the rigorous implementation of the bubble CPAP trial and its follow up roll-out in routine practice. The high level of awareness and sensitivity regarding pneumonia and hypoxaemia management may have reduced severe pneumonia-related deaths in this clinical setting.

We report that hypoxaemia is a powerful predictor of adverse clinical outcomes defined as death during the hospital stay or clinical deterioration requiring referral to a higher-level facility independently or in combination. Other studies have also mentioned hypoxaemia as a strong predictor of mortality in children with WHO-defined pneumonia in lower and middle-income countries [55]. However, the odds of adverse outcomes estimated in our study is relatively higher than the pooled estimate reported by a systematic review conducted by Lazzerini et al. in 2015 [56]. Lazzerini et al. found that, among children with WHO-defined pneumonia, the odds of dying are 5-times higher among those who had hypoxaemia, while it was approximately eight times higher in our study. Variations in the enrolment criteria and severity classifications may explain the difference between these two estimates. Our analysis included only those children who were hospitalised with WHO-defined severe pneumonia, whereas Lazzerini et al. included all children with WHO-defined clinical pneumonia, which may also include non-severe pneumonia who required hospitalisation for other complications. Therefore, the hypoxaemia status among the more severe cases included in our study is expected to have a greater prognostic value in predicting the adverse outcomes. Moreover, Lazzerini et al. conducted sensitivity analysis and reported confidence intervals (2.55–8.61) after adjusting for potential confounders, which marginally overlaps with the confidence interval (5.84-9.86) reported in our study after adjusting for potential confounders and covariates.

In addition to pneumonia, hypoxaemia is common among hospitalised children with several other complications like bronchiolitis, asthma, sepsis, malaria, meningitis, etc. [29,57]. Hence, hypoxaemia can be considered a stand-alone danger sign among young children irrespective of clinical classification, especially in low resource settings. The gold standard for measuring oxygen saturation in the blood is arterial blood gas (ABG) analysis. However, this is invasive, painful, time-consuming and resource intensive. Therefore, it is not helpful for point of care diagnostics and not feasible to integrate into primary care settings with a high volume of patients and limited resources. On the other hand, a pulse oximetry is a non-invasive device used as a point of care diagnostics to obtain a reasonably accurate measurement of blood's oxygen saturation. Appropriate use of pulse oximetry and reliable oxygen sources in developing countries can substantially reduce pneumonia-related mortality [9,10,55]. Duke et al. reported that the introduction of an effective oxygen system could reduce 35% of severe pneumonia deaths, even in resource-poor settings. The locally-made bubble CPAP reduced severe pneumonia mortality by 75% compared to the WHO standard low flow oxygen therapy [21].

The Pneumonia Series published by The Lancet in 2013 outlined the introduction of pulse oximetry as one of the four opportunities for further accelerating progress towards averting the global pneumonia burden in LMICs [58]. Although WHO now recommends routine assessment of SpO_2_ for rapid identification of hypoxaemia and immediate hospitalisation for oxygen therapy with other supportive care, there are significant gaps between policy and actual clinical practice due to insufficient provision for SpO_2_ assessment and inadequate oxygen security in LMIC contexts [17,59-62]. Therefore, urgent attention should be given to improving access, provision, and quality of care regarding overall oxygen security [63,64].

### Strengths and limitations

In this paper, we report the prevalence of hypoxaemia based on retrospective data extracted from individual patient records of routine hospital services. We acknowledge that the quality and oversight during routine clinical assessments are not comparable with controlled environments of clinical trials. However, the icddr,b-Dhaka hospital maintains a high clinical standard by adhering to the national and global guidelines. After the bubble CPAP trial, icddr,b Dhaka hospital introduced routine assessment of hypoxaemia on admission and the use of bubble CPAP for hypoxaemia management as the standard of care. The high level of sensitivity regarding pneumonia and hypoxaemia management may have contributed to the almost universal assessment of hypoxaemia and relevant documentation practices. We also acknowledge the limitation in the availability of detailed clinical information regarding study subjects since the data were obtained from routine clinical records. Therefore, the provision for adjusting for other comorbid conditions such as cyanosis, ability to feed, head nodding, respiratory rate >70/min and unresponsiveness/impaired reusability, crepitations in lung auscultation, nasal flaring as potential confounders [65] and their interaction effects while exploring the effect of hypoxaemia on adverse clinical outcomes was somewhat limited.

The hypoxaemia prevalence reported in this paper is four years of data, which has helped to overcome the potential effect of seasonal variations. Furthermore, the analysis included a reasonably large sample size in reporting hypoxaemia prevalence with an acceptable error margin. We also adjusted for possible confounders in our analysis to give robust estimates of risk factors.

## CONCLUSION

Hypoxaemia is common among children hospitalised with WHO-defined severe pneumonia in Bangladesh. It is also one of the strongest predictors of adverse clinical outcomes, among other background characteristics and history of illnesses. We reinforce the need for pulse oximetry availability and recommend ensuring an effective oxygen delivery system in low- and middle-income countries to avert these preventable deaths.

## Additional material


Online Supplementary Document

## References

[1] Liu L Oza S Hogan D Chu Y Perin J Zhu J Global, regional, and national causes of under-5 mortality in 2000–15: an updated systematic analysis with implications for the Sustainable Development Goals. Lancet. 2016;388:3027-35. 10.1016/S0140-6736(16)31593-8 27839855 PMC5161777

[2] Lim Y-W Steinhoff M Girosi F Holtzman D Campbell H Boer R Reducing the global burden of acute lower respiratory infections in children: the contribution of new diagnostics. Nature. 2006;444:9-18. 10.1038/nature05442 17159890

[3] Oldenburg O Wellmann B Buchholz A Bitter T Fox H Thiem U Nocturnal hypoxaemia is associated with increased mortality in stable heart failure patients. Eur Heart J. 2016;37:1695-703. 10.1093/eurheartj/ehv624 26612581

[4] Wardlaw T, Johansson E, Hodge M. Pneumonia: The Forgotten Killer of Children. UNICEF’s Division of Communication. New York: WHO Press; 2006.

[5] Subhi R Adamson M Campbell H Weber M Smith K Duke T The prevalence of hypoxaemia among ill children in developing countries: a systematic review. Lancet Infect Dis. 2009;9:219-27. 10.1016/S1473-3099(09)70071-4 19324294

[6] Lozano J Epidemiology of hypoxaemia in children with acute lower respiratory infection [oxygen therapy in children]. Int J Tuberc Lung Dis. 2001;5:496-504. 11409574

[7] Reed C Madhi SA Klugman KP Kuwanda L Ortiz JR Finelli L Development of the Respiratory Index of Severity in Children (RISC) score among young children with respiratory infections in South Africa. PLoS One. 2012;7:e27793. 10.1371/journal.pone.0027793 22238570 PMC3251620

[8] Lazzerini M Sonego M Pellegrin MC Hypoxaemia as a mortality risk factor in acute lower respiratory infections in children in low and middle-income countries: systematic review and meta-analysis. PLoS One. 2015;10:e01361660. 10.1371/journal.pone.0136166 26372640 PMC4570717

[9] Enoch AJ English M Shepperd S Does pulse oximeter use impact health outcomes? A systematic review. Arch Dis Child. 2016;101:694-700. 10.1136/archdischild-2015-309638 26699537 PMC4975806

[10] Duke T Wandi F Jonathan M Matai S Kaupa M Saavu M Improved oxygen systems for childhood pneumonia: a multihospital effectiveness study in Papua New Guinea. Lancet. 2008;372:1328-33. 10.1016/S0140-6736(08)61164-2 18708248

[11] McAllister DA Liu L Shi T Chu Y Reed C Burrows J Global, regional, and national estimates of pneumonia morbidity and mortality in children younger than 5 years between 2000 and 2015: a systematic analysis. Lancet Glob Health. 2019;7:e47-57. 10.1016/S2214-109X(18)30408-X 30497986 PMC6293057

[12] National Institute of Population Research and Training (NIPORT), Mitra and Associates, ICF International. Bangladesh Demographic and Health Survey 2017-18. Dhaka, Bangladesh and Calverton, Maryland, USA: 2020.

[13] World Health Organization. UNICEF. End preventable deaths by 2025: The integrated Global Action Plan for Pneumonia and Diarrhoea (GAPPD). France: 2013.

[14] United Nations. Sustainable Development Goals. 2016. Available: http://www.un.org/sustainabledevelopment/. Accessed: 30 March 2020.

[15] Johns Hopkins Bloomberg School of Public Health. Pneumonia & Diarrhea Progress Report 2018. 2018.

[16] World Health Organization. UNICEF. Integrated Management of Childhood Illness (Chart Booklet). Geneva: World Health Organization; 2014.

[17] World Health Organization. Recommendations for management of common childhood conditions: evidence for technical update of pocket book recommendations: newborn conditions, dysentery, pneumonia, oxygen use and delivery, common causes of fever, severe acute malnutrition and supportive care. 2012. 23720866

[18] National Institute of Population Research and Training (NIPORT), Associates for Community and Population Research (ACPR), ICF International. Bangladesh Health Facility Survey 2017. Dhaka, Bangladesh: 2019.

[19] icddr b. icddr,b Annual Report 2019. 2020.

[20] World Health Organization. Pocket book of hospital care for children: guidelines for the management of common childhood illnesses: World Health Organization; 2013. 24006557

[21] Chisti MJ Salam MA Smith JH Ahmed T Pietroni MA Shahunja K Bubble continuous positive airway pressure for children with severe pneumonia and hypoxaemia in Bangladesh: an open, randomised controlled trial. Lancet. 2015;386:1057-65. 10.1016/S0140-6736(15)60249-5 26296950

[22] Stata. Stata. 2021. Available: https://www.stata.com/. Accessed: 3 April 2020.

[23] World Health Organization. Integrated Management of Childhood Illness (IMCI): Chart Booklet: World Health Organization; 2014.

[24] Addo-Yobo E Anh DD El-Sayed HF Fox LM Fox MP MacLeod W Outpatient treatment of children with severe pneumonia with oral amoxicillin in four countries: the MASS study. Trop Med Int Health. 2011;16:995-1006. 10.1111/j.1365-3156.2011.02787.x 21545381 PMC3154370

[25] Atkinson M Lakhanpaul M Smyth A Vyas H Weston V Sithole J A multicentre randomised controlled equivalence trial comparing oral amoxicillin and intravenous benzyl penicillin for community acquired pneumonia in children PIVOT Trial. Thorax. 2007;62:1102-6. 10.1136/thx.2006.074906 17567657 PMC2094276

[26] Bari A Sadruddin S Khan A Khan A Lehri IA Macleod WB Community case management of severe pneumonia with oral amoxicillin in children aged 2–59 months in Haripur district, Pakistan: a cluster randomised trial. Lancet. 2011;378:1796-803. 10.1016/S0140-6736(11)61140-9 22078721 PMC3685294

[27] Orimadegun AE Ogunbosi BO Carson SS Prevalence and predictors of hypoxaemia in respiratory and non-respiratory primary diagnoses among emergently ill children at a tertiary hospital in south western Nigeria. Trans R Soc Trop Med Hyg. 2013;107:699-705. 10.1093/trstmh/trt082 24062524

[28] Ibraheem RM Johnson WB Abdulkarim AA Hypoxaemia in hospitalized under-five Nigerian children with pneumonia. West Afr J Med. 2014;33(1):37-43. 24872265

[29] Graham H Bakare AA Ayede AI Oyewole OB Gray A Peel D Hypoxaemia in hospitalised children and neonates: a prospective cohort study in Nigerian secondary-level hospitals. EClinicalMedicine. 2019;16:51-63. 10.1016/j.eclinm.2019.10.009 31832620 PMC6890969

[30] Bassat Q Lanaspa M Machevo S O’Callaghan-Gordo C Madrid L Nhampossa T Hypoxaemia in Mozambican children< 5 years of age admitted to hospital with clinical severe pneumonia: clinical features and performance of predictor models. Trop Med Int Health. 2016;21:1147-56. 10.1111/tmi.12738 27310711

[31] Tolla HS Letebo M Asemere YA Belete AB Tumbule TC Feleke Z Use of pulse oximetry during initial assessments of children under five with pneumonia: a retrospective cross-sectional study from 14 hospitals in Ethiopia. J Glob Health Rep. 2019;3:e2019016. 10.29392/joghr.3.e2019016 33409377 PMC7771585

[32] Salah ET Algasim SH Mhamoud AS Husian NEOSA Prevalence of hypoxemia in under-five children with pneumonia in an emergency pediatrics hospital in Sudan. Indian J Crit Care Med. 2015;19:203. 10.4103/0972-5229.154549 25878427 PMC4397626

[33] Wandeler G Pauchard J Zangger E Diawara H Gehri M Which clinical signs predict hypoxaemia in young Senegalese children with acute lower respiratory tract disease? Paediatr Int Child Health. 2015;35:65-8. 10.1179/2046905514Y.0000000153 25547179

[34] Mahmud I Das S Khan SH Faruque A Ahmed T Gender disparity in care-seeking behaviours and treatment outcomes for dehydrating diarrhoea among under-5 children admitted to a diarrhoeal disease hospital in Bangladesh: an analysis of hospital-based surveillance data. BMJ Open. 2020;10:e038730. 10.1136/bmjopen-2020-038730 32883737 PMC7473626

[35] Naheed A Breiman RF Islam MS Saha SK Tabassum Naved R Disparities by sex in care-seeking behaviors and treatment outcomes for pneumonia among children admitted to hospitals in Bangladesh. PLoS One. 2019;14:e0213238. 10.1371/journal.pone.0213238 30845206 PMC6405050

[36] Nair H Simões EA Rudan I Gessner BD Azziz-Baumgartner E Zhang JSF Global and regional burden of hospital admissions for severe acute lower respiratory infections in young children in 2010: a systematic analysis. Lancet. 2013;381:1380-90. 10.1016/S0140-6736(12)61901-1 23369797 PMC3986472

[37] Nair H, Campbell H, Park J, Brondi L, Shi T, Olsson S, et al. Common childhood infections and gender inequalities: a systematic review. UNICEF Maternal, Newborn and Child Health Working Papers New York, NY. 2015.

[38] Chisti MJ Duke T Robertson CF Ahmed T Faruque AS Ashraf H Clinical predictors and outcome of hypoxaemia among under-five diarrhoeal children with or without pneumonia in an urban hospital, Dhaka, Bangladesh. Trop Med Int Health. 2012;17:106-11. 10.1111/j.1365-3156.2011.02890.x 21951376

[39] Angoulvant F Levy C Grimprel E Varon E Lorrot M Biscardi S Early impact of 13-valent pneumococcal conjugate vaccine on community-acquired pneumonia in children. Clin Infect Dis. 2014;58:918-24. 10.1093/cid/ciu006 24532543

[40] Tregnaghi MW Sáez-Llorens X López P Abate H Smith E Pósleman A Efficacy of pneumococcal nontypable Haemophilus influenzae protein D conjugate vaccine (PHiD-CV) in young Latin American children: a double-blind randomized controlled trial. PLoS Med. 2014;11:e1001657. 10.1371/journal.pmed.1001657 24892763 PMC4043495

[41] Simonsen L Taylor RJ Schuck-Paim C Lustig R Haber M Klugman KP Effect of 13-valent pneumococcal conjugate vaccine on admissions to hospital 2 years after its introduction in the USA: a time series analysis. Lancet Respir Med. 2014;2:387-94. 10.1016/S2213-2600(14)70032-3 24815804

[42] Jain S Williams DJ Arnold SR Ampofo K Bramley AM Reed C Community-acquired pneumonia requiring hospitalization among US children. N Engl J Med. 2015;372:835-45. 10.1056/NEJMoa1405870 25714161 PMC4697461

[43] Rhedin S Lindstrand A Hjelmgren A Ryd-Rinder M Öhrmalm L Tolfvenstam T Respiratory viruses associated with community-acquired pneumonia in children: matched case–control study. Thorax. 2015;70:847-53. 10.1136/thoraxjnl-2015-206933 26077969

[44] Shi T McAllister DA O’Brien KL Simoes EA Madhi SA Gessner BD Global, regional, and national disease burden estimates of acute lower respiratory infections due to respiratory syncytial virus in young children in 2015: a systematic review and modelling study. Lancet. 2017;390:946-58. 10.1016/S0140-6736(17)30938-8 28689664 PMC5592248

[45] Jackson S Mathews KH Pulanić D Falconer R Rudan I Campbell H Risk factors for severe acute lower respiratory infections in children–a systematic review and meta-analysis. Croat Med J. 2013;54:110-21. 10.3325/cmj.2013.54.110 23630139 PMC3641871

[46] Usen S Weber M Mulholland K Jaffar S Oparaugo A Omosigho C Clinical predictors of hypoxaemia in Gambian children with acute lower respiratory tract infection: prospective cohort study. BMJ. 1999;318:86-91. 10.1136/bmj.318.7176.86 9880280 PMC27680

[47] Ayieko P Graham S In children aged 2-59 months with pneumonia, which clinical signs best predict hypoxaemia? J Trop Pediatr. 2006;52:307-10. 10.1093/tropej/fml036 16943216

[48] Ahmed HM Mitchell M Hedt B National implementation of Integrated Management of Childhood Illness (IMCI): policy constraints and strategies. Health Policy. 2010;96:128-33. 10.1016/j.healthpol.2010.01.013 20176407

[49] Walter ND Lyimo T Skarbinski J Metta E Kahigwa E Flannery B Why first-level health workers fail to follow guidelines for managing severe disease in children in the Coast Region, the United Republic of Tanzania. Bull World Health Organ. 2009;87:99-107. 10.2471/BLT.08.050740 19274361 PMC2636200

[50] Arifeen SE Bryce J Gouws E Baqui A Black R Hoque D Quality of care for under-fives in first-level health facilities in one district of Bangladesh. Bull World Health Organ. 2005;83:260-7. 15868016 PMC2626213

[51] Lange S Mwisongo A Mæstad O Why don’t clinicians adhere more consistently to guidelines for the Integrated Management of Childhood Illness (IMCI)? Soc Sci Med. 2014;104:56-63. 10.1016/j.socscimed.2013.12.020 24581062

[52] Hazir T Begum K El Arifeen S Khan AM Huque MH Kazmi N Measuring coverage in MNCH: a prospective validation study in Pakistan and Bangladesh on measuring correct treatment of childhood pneumonia. PLoS Med. 2013;10:e1001422. 10.1371/journal.pmed.1001422 23667339 PMC3646205

[53] Lazzerini M Seward N Lufesi N Banda R Sinyeka S Masache G Mortality and its risk factors in Malawian children admitted to hospital with clinical pneumonia, 2001–12: a retrospective observational study. Lancet Glob Health. 2016;4:e57-68. 10.1016/S2214-109X(15)00215-6 26718810 PMC5495601

[54] Ramachandran P Nedunchelian K Vengatesan A Suresh S Risk factors for mortality in community-acquired pneumonia among children aged 1–59 months admitted in a referral hospital. Indian Pediatr. 2012;49:889-95. 10.1007/s13312-012-0221-3 22791667

[55] Duke T Mgone J Frank D Hypoxaemia in children with severe pneumonia in Papua New Guinea [oxygen therapy in children]. Int J Tuberc Lung Dis. 2001;5:511-9. 11409576

[56] Lazzerini M Sonego M Pellegrin MC Hypoxaemia as a mortality risk factor in acute lower respiratory infections in children in low and middle-income countries: systematic review and meta-analysis. PLoS One. 2015;10:e0136166. 10.1371/journal.pone.0136166 26372640 PMC4570717

[57] Rahman AE Iqbal A Hoque DE Moinuddin M Zaman SB Rahman QS-u Managing neonatal and early childhood syndromic sepsis in sub-district hospitals in resource poor settings: improvement in quality of care through introduction of a package of interventions in rural Bangladesh. PLoS One. 2017;12:e0170267. 10.1371/journal.pone.0170267 28114415 PMC5256881

[58] Chopra M Mason E Borrazzo J Campbell H Rudan I Liu L Ending of preventable deaths from pneumonia and diarrhoea: an achievable goal. Lancet. 2013;381:1499-506. 10.1016/S0140-6736(13)60319-0 23582721

[59] Fuchs A Bielicki J Mathur S Sharland M Van Den Anker JN Reviewing the WHO guidelines for antibiotic use for sepsis in neonates and children. Paediatr Int Child Health. 2018;38:S3-15. 10.1080/20469047.2017.1408738 29790842 PMC6176768

[60] Arifeen SE Bryce J Gouws E Baqui A Black R Hoque D Quality of care for under-fives in first-level health facilities in one district of Bangladesh. Bull World Health Organ. 2005;83:260-7. 15868016 PMC2626213

[61] Anwar I Kalim N Koblinsky M Quality of obstetric care in public-sector facilities and constraints to implementing emergency obstetric care services: evidence from high-and low-performing districts of Bangladesh. J Health Popul Nutr. 2009;27:139. 10.3329/jhpn.v27i2.3327 19489412 PMC2761772

[62] Chowdhury S Hossain SA Halim A Assessment of quality of care in maternal and newborn health services available in public health care facilities in Bangladesh. Bangladesh Med Res Counc Bull. 2009;35:53-6. 10.3329/bmrcb.v35i2.3044 20120780

[63] Duke T Graham S Cherian M Ginsburg A English M Howie S Oxygen is an essential medicine: a call for international action [Unresolved issues]. Int J Tuberc Lung Dis. 2010;14:1362-8. 20937173 PMC2975100

[64] Bhutta ZA Das JK Walker N Rizvi A Campbell H Rudan I Interventions to address deaths from childhood pneumonia and diarrhoea equitably: what works and at what cost? Lancet. 2013;381:1417-29. 10.1016/S0140-6736(13)60648-0 23582723

[65] Zhang L Mendoza-Sassi R Santos J Lau J Accuracy of symptoms and signs in predicting hypoxaemia among young children with acute respiratory infection: a meta-analysis. Int J Tuberc Lung Dis. 2011;15:317-25. 21333097

