# Supplementary material for: Hypoxaemia prevalence and its adverse clinical outcomes among children hospitalised with WHO-defined severe pneumonia in Bangladesh
Source: J Glob Health. 2021 Sep 11;11:04053. doi: 10.7189/jogh.11.04053 (PMC8442579; doi:10.7189/jogh.11.04053)
Supplement: Online Supplementary Document [file jogh-11-04053-s001.pdf]

Supplementary Materials

Figure S1: Hypoxaemia prevalence among children aged 2-59 months admitted to icddr,b Dhaka Hospital with WHO-defined severe pneumonia between 2014-17, presented in percentage by age groups; N=2646.

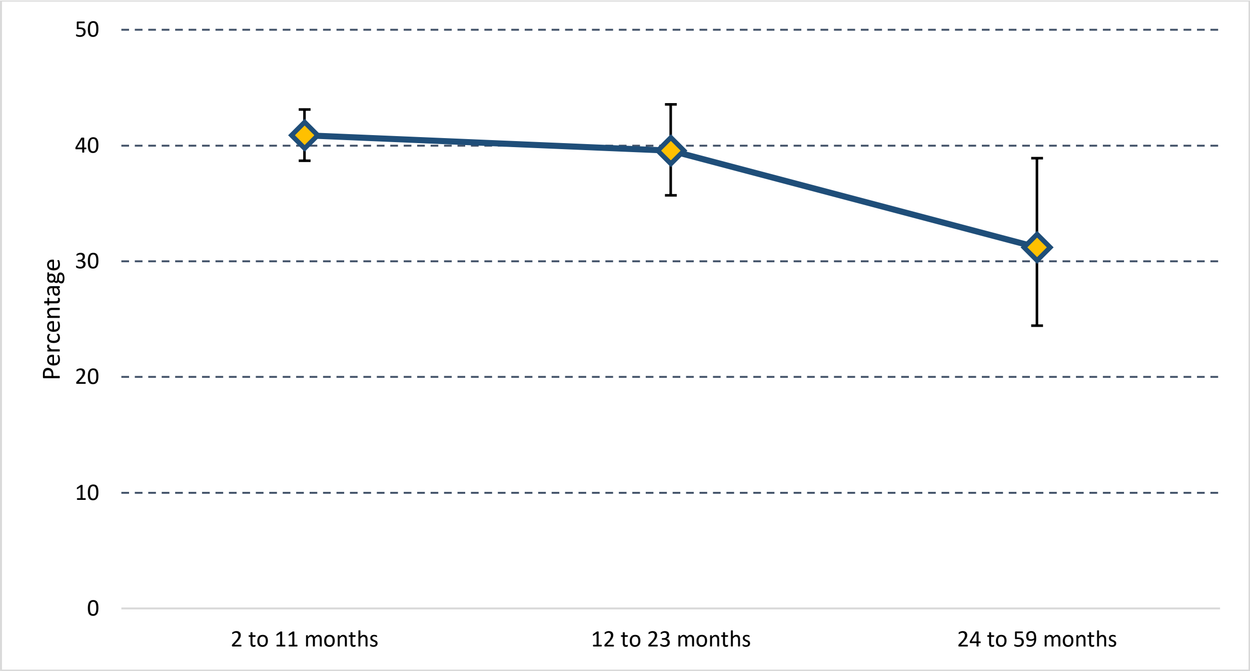

Figure S2: Distribution of hospital outcomes of children aged 2-59 months admitted to icddr,b-Dhaka Hospital with WHO-defined clinical pneumonia between 2014-17, presented in percentage by hypoxaemia status on admission.

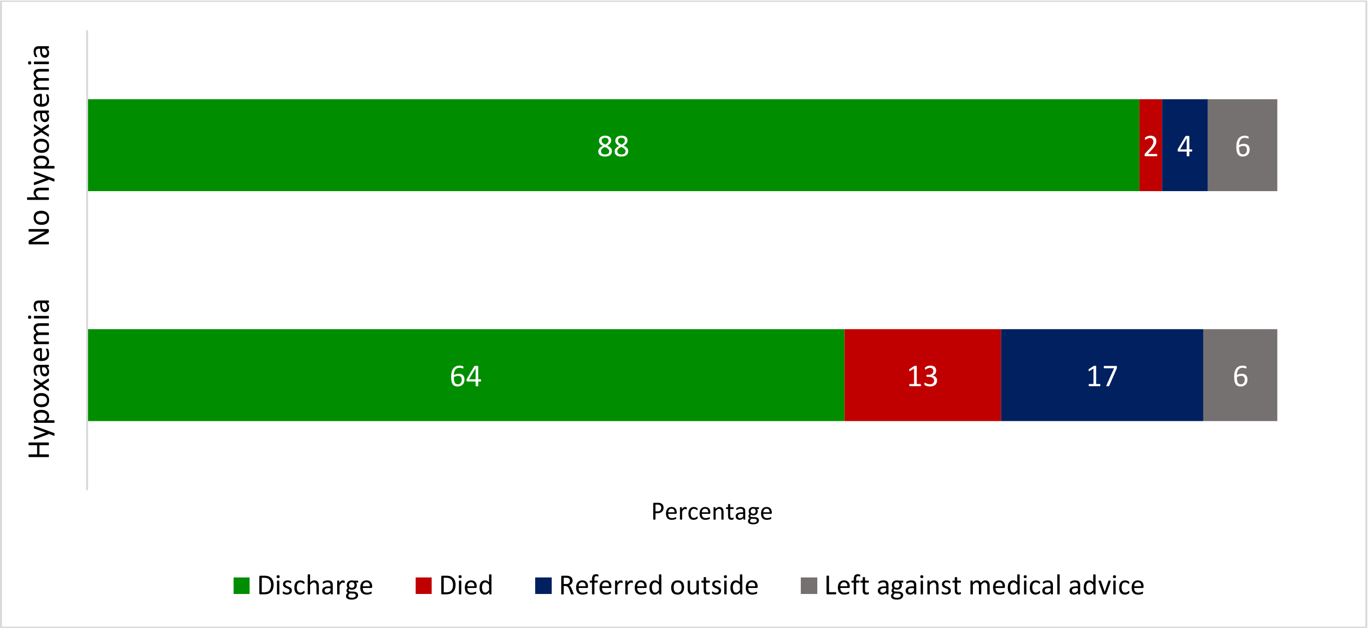

Figure S3: Trend in the case fatality rates of children aged 2-59 months admitted to icddr,b-Dhaka Hospital with WHO-defined clinical pneumonia between 2014-17, presented in percentage by hypoxaemia status on admission and disaggregated by the year.

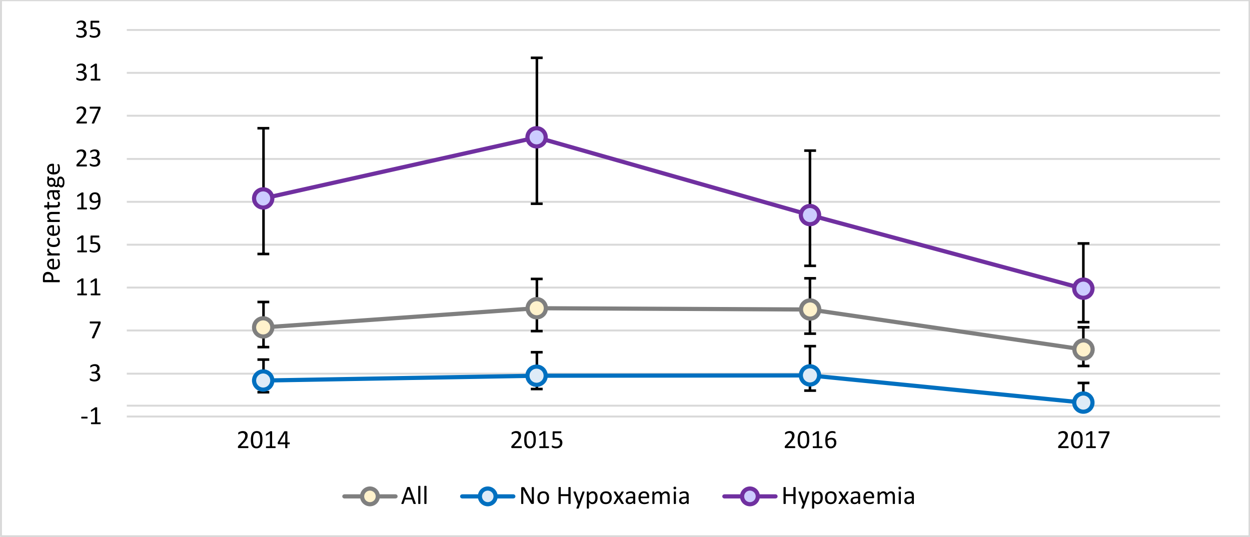

Figure S4: Risk of adverse hospital outcomes among children aged 2-59 months admitted to icddr,b Dhaka Hospital with WHO-defined severe pneumonia and hypoxaemia between 2014-17, presented in adjusted odds ratio; N=2646.

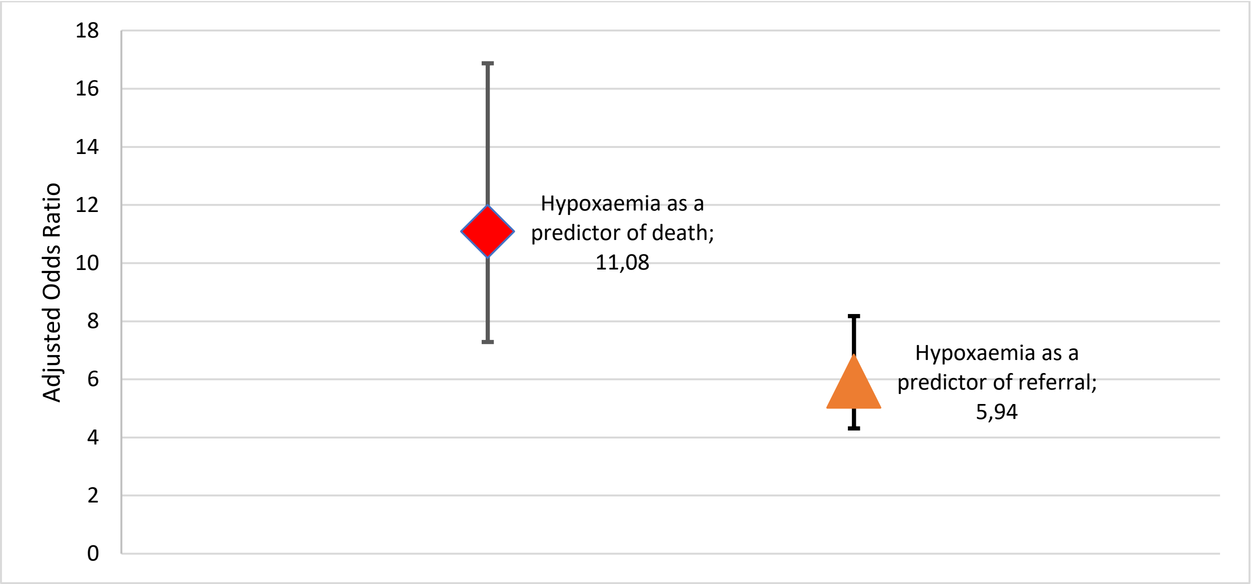

Table S1: Predictors of adverse outcomes as either death during hospital stay or referral to higher level facility for clinical deterioration

Background Characteristics	N	%	OR	P	Lower	Upper	AOR	P	Lower	Upper
**Age**										
2 - 11 months	308	16.3								
12 months - 59 months	102	13.6	0.80	0.08	0.63	1.03	0.83	0.16	0.64	1.08
**Sex**										
Male	238	13.9								
Female	172	18.3	1.39	0.00	1.12	1.72	1.23	0.08	0.97	1.55
**H/O Fever**										
No fever	155	14.1								
0-1 days	48	20.1	1.55	0.02	1.08	2.22	1.35	0.14	0.91	1.99
2-6 days	156	15	1.06	0.63	0.83	1.35	1.00	0.99	0.77	1.29
7 or more days	51	19.2	1.46	0.04	1.03	2.00	1.53	0.03	1.05	2.25
**H/O Cough or difficulty breathing**										
No respiratory distress	283	14.5								
0-48 hours	81	21.1	1.56	0.00	1.18	2.05	1.20	0.23	0.89	1.62
More than 48 hours	46	14.7	1.00	0.99	0.71	1.41	0.88	0.50	0.61	1.27
**Severe Acute Malnutrition**										
No	205	18.4								
Yes	205	13.4	0.72	0.00	0.58	0.89	1.26	0.06	0.99	1.60
**Hypoxaemia**										
No	91	5.7								
Yes	319	30.1	7.30	0.00	5.68	9.37	7.59	0.00	5.84	9.86
**Total**	410	15.5								

Table S2: Predictors of death during hospital stay

Background Characteristics	N	%	OR	P	Lower	Upper	AOR	P	Lower	Upper
**Age**										
2 - 11 months	128	6.8								
12 months - 59 months	41	5.5	0.78	0.18	0.54	1.12	0.84	0.36	0.57	1.23
**Sex**										
Male	91	5.3								
Female	78	8.3	1.65	0.00	1.20	2.26	1.45	0.03	1.04	2.02
**H/O Fever**										
No fever	70	6.4								
0-1 days	19	7.9	1.36	0.26	0.80	2.31	1.28	0.39	0.73	2.25
2-6 days	57	5.5	0.86	0.41	0.60	1.23	0.84	0.36	0.57	1.23
7 or more days	23	8.6	1.46	0.14	0.89	2.40	1.50	0.13	0.89	2.56
**H/O Cough or difficulty breathing**										
No respiratory distress	124	6.4								
0-48 hours	21	5.5	0.92	0.73	0.57	1.49	0.74	0.25	0.45	1.23
More than 48 hours	24	7.7	1.19	0.45	0.76	1.89	1.05	0.84	0.65	1.71
**Severe Acute Malnutrition**										
No	75	6.7								
Yes	94	6.1	0.90	0.53	0.66	1.24	1.65	0.01	1.17	2.33
**Hypoxaemia**										
No	30	1.9								
Yes	139	13.1	9.64	0.00	6.43	14.46	11.08	0.00	7.28	16.87
**Total**	**169**	**6.4**								

Table S3: Predictors of referral to higher level facility for clinical deterioration

Background Characteristics	N	%	OR	P	Lower	Upper	AOR	P	Lower	Upper
**Age**										
2 - 11 months	180	9.5								
12 months - 59 months	61	8.1	0.82	0.21	0.61	1.12	0.84	0.29	0.61	1.16
**Sex**										
Male	147	8.6								
Female	94	10	1.23	0.14	0.93	1.62	1.09	0.54	0.82	1.46
**H/O Fever**										
No fever	85	7.7								
0-1 days	29	12.1	1.71	0.02	1.09	2.68	1.36	0.21	0.85	2.19
2-6 days	99	9.5	1.23	0.19	0.91	1.67	1.13	0.45	0.82	1.56
7 or more days	28	10.5	1.46	0.10	0.93	2.30	1.56	0.07	0.97	2.52
**H/O respiratory distress**										
No respiratory distress	159	8.2								
0-48 hours	60	15.6	2.05	0.00	1.48	2.83	1.54	0.01	1.09	2.18
More than 48 hours	22	7	0.85	0.51	0.54	1.36	0.75	0.24	0.46	1.21
**Severe Acute Malnutrition**										
No	130	11.6								
Yes	111	7.3	0.62	0.00	0.47	0.80	1.07	0.65	0.80	1.44
**Hypoxaemia**										
No	61	3.8								
Yes	180	17	6.14	0.00	4.53	8.33	5.94	0.00	4.31	8.17
**Total**	**241**	**9.1**								
